# Role of Microbiota in Viral Infections and Pathological Progression

**DOI:** 10.3390/v14050950

**Published:** 2022-05-01

**Authors:** Taketoshi Mizutani, Aya Ishizaka, Michiko Koga, Takeya Tsutsumi, Hiroshi Yotsuyanagi

**Affiliations:** 1 Department of Computational Biology and Medical Sciences, Graduate School of Frontier Sciences, The University of Tokyo, Chiba 277-8562, Japan; 2 Division of Infectious Diseases, Advanced Clinical Research Center, the Institute of Medical Science, The University of Tokyo, Tokyo 108-8639, Japan; ishizaka@ims.u-tokyo.ac.jp (A.I.); michiko@ims.u-tokyo.ac.jp (M.K.); tsutsumi@ims.u-tokyo.ac.jp (T.T.); yotsudid@ims.u-tokyo.ac.jp (H.Y.); 3 Department of Infectious Diseases and Applied Immunology, IMSUT Hospital of Institute of Medical Science, The University of Tokyo, Tokyo 108-8639, Japan

**Keywords:** infectious disease, microbiota, microbiome, dysbiosis, virome, SARS-CoV-2, COVID-19, HIV, hepatitis virus, HPV

## Abstract

Viral infections are influenced by various microorganisms in the environment surrounding the target tissue, and the correlation between the type and balance of commensal microbiota is the key to establishment of the infection and pathogenicity. Some commensal microorganisms are known to resist or promote viral infection, while others are involved in pathogenicity. It is also becoming evident that the profile of the commensal microbiota under normal conditions influences the progression of viral diseases. Thus, to understand the pathogenesis underlying viral infections, it is important to elucidate the interactions among viruses, target tissues, and the surrounding environment, including the commensal microbiota, which should have different relationships with each virus. In this review, we outline the role of microorganisms in viral infections. Particularly, we focus on gaining an in-depth understanding of the correlations among viral infections, target tissues, and the surrounding environment, including the commensal microbiota and the gut virome, and discussing the impact of changes in the microbiota (dysbiosis) on the pathological progression of viral infections.

## 1. Introduction

Viruses that are pathogenic to humans include those of the respiratory system (e.g., adenovirus, influenza, and severe acute respiratory syndrome coronavirus-2 (SARS-CoV-2)); colon (e.g., enteroviruses, rotavirus, and norovirus); liver (e.g., hepatitis virus); spinal cord (e.g., poliovirus); cervix (e.g., human papillomavirus (HPV)); and white blood cells (e.g., human immunodeficiency virus (HIV) and human T-cell leukemia virus (HTLV)). A viral infection is initiated by the interaction of the viral capsid or envelope glycoproteins with host cell-specific receptors. After entering the target cell, the sequence of viral nucleic acid amplification, generation of viral particles, and exit from the cell constitutes the viral life cycle [1]. During this process, some viruses, such as HIV and hepatitis viruses, incorporate their viral genome into the host cell genome, thereby persistently infecting the host; however, most viruses are rapidly eliminated from the body, owing to the host’s immune response, and remain only in transient infections. Viruses cannot replicate and multiply independently; they use host cells as scaffolds and require their transcriptional machinery to make multiple viral copies. The involvement of symbiotic microorganisms in the establishment of an infection and the progression of a disease associated with this process should not be underestimated.

**Figure 1:**
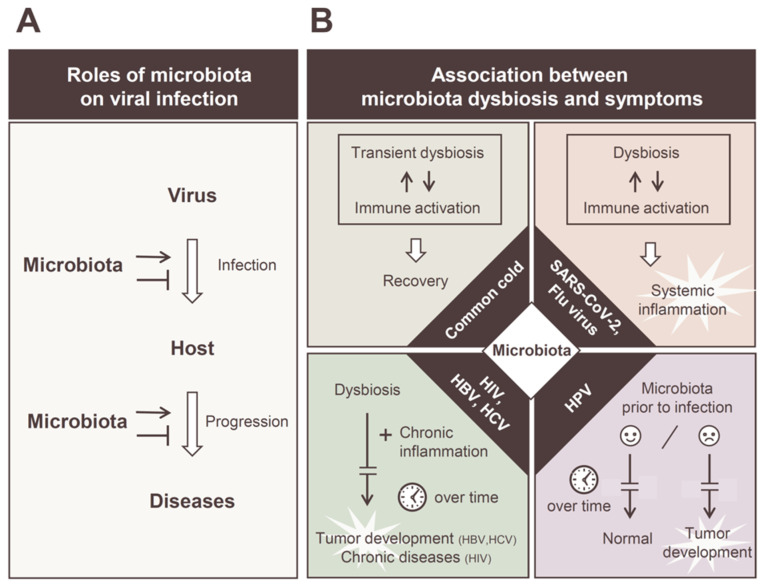
Diverse roles of commensal microbiota in viral infections. (**A**) Schematic representation of the bacterial intervention in viral infection and pathogenesis. The commensal microbiota can interact with invading viruses and play enhancing or suppressive roles to viral infections. The composition of the intestinal microbiota can promote or suppress disease progression. (**B**) Correlations between microbiota dysbiosis and disease progression in individual viral infections. Transient dysbiosis of the intestinal microbiota occurs even in the common cold and mild cases of influenza and SARS-CoV-2 infections, which is considered an additional reaction to the normal immune response (upper left). It is suggested that alterations in the intestinal microbiota are involved in the induction of a cytokine storm in severe cases of influenza and SARS-CoV-2 infection (upper right). In chronic viral infections, such as those of HIV, HBV, and HCV, intestinal microbiota and chronic inflammation facilitate the disease progression over time (lower left). The vaginal microbiota before HPV infection has a significant impact on the development of HPV cervical cancer (lower right).

Recent studies revealed that the symbiotic relationship between the host and indigenous microbiota plays an important role in establishing viral infections. First, the presence of commensal bacteria in the mucosa is a physical barrier to cell adhesion, which is the first step in establishing a viral infection (Figure 1A) [1]. The secretion of soluble factors by some intestinal bacteria and continuous stimulation of the host immune system by bacterial-derived factors contribute to the antiviral effect [2]. However, the molecular mechanisms by which viruses overcome or exploit these barriers to establish an infection are of great academic interest. Many studies have been conducted regarding this, because this understanding can help develop “controlling microbiota” strategies to confer protection against viral infections. Moreover, crosstalk, such as physical or material transfers between the host cell and the microbiota on the cell epithelium, is considered important in the development of viral diseases. This also affects intracellular events such as the regulation of the gene expression of infected viruses. In addition, the susceptibility of indigenous microbiota varies greatly, depending on the target tissues of the virus, and the pathogenesis that develops also differs, depending on the virus species; therefore, understanding the environmental factors of each tissue is important for clarifying the establishment and development of a viral infection (Figure 1B). In some viral infections, such as HTLV, the relationship between the pathogenicity and microbiota is still uncertain. It is also becoming evident that an individual’s clinical background, including any underlying conditions, which is becoming apparent in the coronavirus disease (COVID-19) pandemic, can affect the prognosis of viral infections [3,4,5]. Thus, the profile of the microbiota in the healthy stage before infection also has a significant impact on the disease prognosis. 

Microorganisms that live symbiotically in humans form various colonies, mainly in the skin, oral cavity, upper respiratory tract, intestinal tract, and reproductive organs [6]. Regarding the relationship between viral infection and symbiotic microorganisms, viral infection, replication, and pathogenesis are influenced by symbiotic microorganisms living in the vicinity of target tissues. HPV, which infects the oral cavity and genital tract, and noroviruses and rotaviruses, which infect the intestinal epithelium, are representative examples of this relationship. However, changes in the distal intestinal microbiota are observed in respiratory viruses and hepatitis viruses that infect the liver. Thus, it has been suggested that the microbiota acts systemically rather than locally in viral infections, and “gut–multiple organ” axis theories have been proposed [7]. In other words, the symbiotic microbiota can be regarded as an important factor governing the clinical outcomes during viral infection.

The concept of controlling the microbiota is being recognized as a new antiviral strategy to prevent the onset of viral infections and severe prognosis following an infection. In terms of how to control the intestinal microbiota in our daily lives, moderate exercise is suggested to have beneficial effects on health, such as reducing inflammation and intestinal permeability and improving the body composition [8]. In addition, mental health initiatives such as stress reduction and improvement of the oral environment are also effective in preserving the intestinal environment [9,10]. As the concept of preventive medicine grows in importance, this assertion will gain more credibility. 

In this review, we outline the correlations among viral infection, target tissues, and the surrounding environment, including the indigenous microbiota of target tissues, with the aim of understanding the role of the indigenous microbiota in viral infections. Although there are various types of viruses that infect humans, this review focuses on five groups of viruses that have been widely reported to be associated with the microbiota: diarrhea viruses (norovirus and rotavirus); respiratory viruses such as influenza and SARS-CoV-2; hepatitis viruses (A, B, C, and E); immunodeficiency virus (HIV); and cervical cancer virus (HPV). In particular, this review clarifies the relationship between the virus and the microbiota, focusing on the differences in the infected tissues, which will be useful for understanding the pathogenesis of infectious diseases that vary among the tissues and the involvement of indigenous bacteria. 

## 2. Commensal Microbiota and Human Health

In humans, there are a vast number and variety of microorganisms, including bacteria, fungi, and viruses [6]. Of these, bacteria, which are by far the most numerous, have been the focus of much research because of their importance to human health. Microorganisms colonize at a range of sites in the body, including the skin, oral cavity, upper respiratory tract, gastrointestinal tract, and reproductive organs. Among them, representative intestinal bacteria are classified into three types according to their actions. Beneficial bacteria are those that are effective in maintaining health and preventing aging, such as by aiding digestion and absorption and enhancing immunity [7,11]. Typical bacteria include *bifidobacterium* and lactic acid bacteria. Conversely, harmful bacteria are considered to have adverse effects on the body. Typical examples include Welsh bacillus, staphylococci, and toxic *E. coli*. In addition, opportunistic bacteria are those that are harmless when in healthy quantities but increase in the intestine when the body is weakened (occurrence of opportunistic infections); typical examples are *Bacteroides*, *E. coli* (non-toxic strains), and *Streptococcus*. The presence of symbiotic bacteria on mucosal surfaces has been reported to be important for immune homeostasis and the immune response [12,13]. In particular, the mucosa is a route of entry into the cell from outside, and the microbiota present in the gut mucosa constantly stimulates immune cells and promotes the maturation of secondary lymphoid tissues in the gastrointestinal tract that are responsible for the defense of the intestinal mucosa [14]. This system leads to a rapid immune response upon the invasion of foreign substances (viruses and parasites) from outside. Interestingly, wild mice have a bacterial microbiota that has evolved under greater evolutionary pressures, including infection and naturally occurring inflammatory immune stimuli, compared with laboratory mice, which have reported an improved fitness [15,16]. Many previous reports have shown that dysbiosis is associated with pathogenesis in autoimmune diseases, such as inflammatory bowel disease [17]. Given the above evidence, the balance of the microbiota is an important factor in immune homeostasis and pathogenesis [18].

## 3. Acute Gastroenteritis

### 3.1. Acute Gastroenteritis Virus and Intestinal Disorders

Several viruses, bacteria, and parasites have been identified as causes of acute gastroenteritis, with norovirus accounting for approximately 20% of cases globally, rotavirus being one of the leading causes of infant mortality worldwide [19]. The rotavirus infects the epithelium of the small intestine and causes rapid dehydration with disruption of the small intestinal epithelial cells; it is one of the leading causes of death among infants under 5 years of age in developing countries. Sapovirus, astrovirus, and adenovirus are also causative agents of acute gastroenteritis. Gastroenteritis viruses, such as rotavirus and norovirus, are transmitted to healthy individuals through water or food contaminated with the vomit or feces of infected persons, and their infections are frequently reported in developing countries and unsanitary environments. 

### 3.2. Role of Microbiota in the Establishment of Acute Gastroenteritis Virus Infection

Acute gastroenteritis generally causes abdominal pain with diarrhea. This is accompanied by marked changes in the intestinal microbiota due to the infection. Noroviruses are reported in animal studies and in vitro studies on the interaction between the virus and the microbiota during infection [20]. In vitro experiments have shown that human noroviruses can infect B cells in the presence of bacteria coated with histo-blood group antigens. This suggests an infection mechanism involving bacteria attached to the intestinal epithelium. The removal of microbiota by antibiotic treatment prevents persistent infection with the murine norovirus [21]. There are similar reports on the poliovirus, reovirus, murine mammary tumor virus, and rotavirus [21,22,23,24]. It is also reported that lipopolysaccharides (LPSs) enhance the stability and infectivity of poliovirus particles [25,26]. These findings suggest that some microorganisms of the intestinal microbiota promote enteroviral infections, which has been reviewed extensively elsewhere [27,28,29]. In contrast, experiments with immunocompromised mice showed that segmented filamentous bacteria have protective properties against rotavirus infection independent of antiviral activities of immune cells [30]. Furthermore, there are reports suggesting that microbiota inhibit the cell adhesion of noroviruses during in vitro experiments [31,32]. The diversity of the intestinal bacterial layer decreases after norovirus or rotavirus infection [33], and the composition of the microbiota is different in patients with diarrhea in Ghana [34]. These observations may be explained by the differences in interactions with the microbiota in viral infections.

### 3.3. Commensal Microbiota and Antiviral Immune Response

It has been shown that the presence of gut microbiota is important as an immune control through type I interferon, which rapidly induces an innate immunity against the virus. A study using mice reported that interferon induction is attenuated when intestinal bacteria are depleted by antibiotics [35]. Secretory immunoglobulin A secreted by the intestinal mucosa is also considered an effective mucosal barrier for virus elimination, but the amount of IgA is low in germ-free mice, and commensal bacteria may be involved in the steady-state secretion of IgA and the rapid immune response [36]. Furthermore, a norovirus infection significantly alters the human intestinal microbiota, suggesting that the intestinal microbiota is closely involved in the immune response to the norovirus [37]. Based on these findings, abnormalities of indigenous bacteria observed immediately after an infection can be considered a side effect of the normal immune response. Diarrheal symptoms caused by abnormalities in the intestinal microbiota can also be considered a series of immune responses for eliminating pathogens from the body. 

Although there is a vaccine for the rotavirus, it is not widely available globally [38]. Infectious diarrhea is an urgent problem, especially in developing countries, and the development of a norovirus vaccine is desirable, along with its widespread use. Probiotics are expected to regulate the composition of the intestinal microbiota, enhance the intestinal barrier function, and control infectious diarrhea [39,40]. The discovery of effective probiotic strains and combinations of strains is important, but it is also necessary to determine their efficacy, because indigenous bacteria vary among countries [41].

## 4. Upper Respiratory Tract Infections

### 4.1. Upper Respiratory Tract Infections and Gastrointestinal Disease

Several viruses, including influenza and adenovirus, are known to cause upper respiratory tract infections, which are mainly characterized by upper respiratory tract inflammation and may have symptoms, including sore throat, cough, and fever. Upper respiratory tract infections are often accompanied by gastrointestinal disorders, such as abdominal pain and diarrhea. This is explained by the gut–lung axis, which is a physiological connection between the intestines and lungs [42]. It is known that the airways and intestinal tract interact with each other, and changes in one can affect the other. Changes in the gut environment are associated with the pathogenesis of respiratory diseases, which may involve bronchial asthma and microbiota [43]. Thus, some changes in the microbiota are reported after an influenza infection (H1N1 and H7N9) [44,45]. 

Some upper respiratory tract infections are relatively more prevalent in children than in adults. This may be because the composition of the intestinal microbiota is different between children and adults [46]. The most recent report showed that disruption of the respiratory microbiota in the early postnatal period increases the susceptibility to respiratory tract infections [47]. Although few clinical studies have described the relationship between adenoviruses and astroviruses that cause diarrhea-like symptoms and affect the intestinal microbiota, dysbiosis after infections has been reported in animal studies [48,49]. Age-dependent underdevelopment of the immune system and gut microbiota may be involved; experiments involving the astrovirus in bats reported that the abundance of bacterial taxa characteristic of a healthy microbiota is markedly reduced in young astrovirus-positive bats. 

### 4.2. Influence of Commensal Microbiota in Upper Respiratory Tract Infection

Another interesting finding is that some microorganisms of the indigenous microbiota act defensively against viral infections in upper respiratory tract infections [50,51,52,53,54]. A recent report showed that the susceptibility to influenza A (H3N2) and B infections is associated with indigenous nasal and pharyngeal microbiota [55]. Interferon induction related to the innate immunity against the genus *Staphylococcus* in the nasal cavity is reported to be a molecular mechanism underlying the protective function of indigenous microbiota against viruses [56]. Currently, there is growing evidence that changes in the commensal microbiota affect the prognosis of viral diseases [57], including recent studies involving COVID-19, although the detailed molecular mechanisms are not clear. SARS-CoV-2 infection is associated with hypertension, diabetes, heart disease, and other underlying diseases; it is also associated with rapid inflammation, including cytokine storm formation [58,59]. Variations in the gut microbiota, which play a role in immune homeostasis, are suggested to be involved in this severe disease, according to several reports [60,61,62,63].

### 4.3. SARS-CoV-2 Infection and Alteration of the Microbiota 

Changes in the diversity of the intestinal microbiota are reported immediately after SARS-CoV-2 infection [44,64,65,66,67,68]. The intestinal microbiota of the patients is observed to change gradually immediately after the onset of the disease compared with that of healthy subjects, and the changes peaked at 2 to 3 weeks [64]. This was attributed to the decrease in the number of Ruminococcaceae and Lachnospiraceae bacteria [66] or a decrease in the bacteria of the phylum Firmicutes, mainly the genus *Faecalibacterium*; an increase in some opportunistic bacteria was also reported [44,67,68]. While all the above results have been reported in China and Japan, elevated levels of the genera associated with gastrointestinal disease (*Campylobacter* and *Klebsiella*) have been observed in African American patients in the United States [65]. Furthermore, dysbiosis in the gut microbiota (decrease in bacteria producing short-chain fatty acids) after an infection in animal models using hamsters has also been reported [69]. Changes in the oral microbiota after infection with SARS-CoV-2 have also been reported in many studies [70,71,72,73,74,75]. Ma et al. reported that an analysis of oropharyngeal swab specimens showed that changes in the oral microbiota after infection with SARS-CoV-2 elicited an inflammatory response and affected the severity of the disease [71]. Additionally, a comparative analysis of the gut microbiota and oral bacteria between influenza patients and COVID-19 patients showed different compositions of the microbiota [44,71]. 

### 4.4. Understanding of the Microbiota in Therapeutic Intervention for COVID-19

The dysbiosis observed immediately after infection may be due to immune responses in the mucosal epithelium, including attacks of indigenous bacteria by innate immunity. Thus, the imbalanced microbiota may cause a temporary increase in opportunistic infections. These observations are transient, and the involvement of the microbiota in disease progression has not been fully established; however, positive correlations between inflammation [64,67] or disease severity and the gut microbiota have been reported [60,67,76]. Recent reports described changes in the fungal microbiota of the lungs of patients with COVID-19 [77]. In addition, an increasing number of reports using a shotgun analysis of patients’ stool specimens described the correlation among the changes in the gut microbiota, immune responses, and metabolic pathway [67,78,79,80]. Compared with healthy individuals, patients have increased levels of blood markers such as inflammatory cytokines and C-reactive proteins after an infection [67] and multiple altered pathway modules in metabolic pathways in association with a decrease in immune-regulatory bacteria, such as those from the phylum Firmicutes [78,79,80]. Based on these reports, future studies should evaluate the longitudinal and cross-sectional analyses of patients’ intestinal microbiota during disease progression. It is also suggested that the identification of bacteria involved in a severe disease and an understanding of the metabolic functions of these bacteria may serve as a molecular basis for the COVID-19 pathogenesis. In COVID-19, the pathogenicity is highly dependent on the underlying disease of the individual, and the composition of the gut microbiota before onset may be important. In a recent cross-sectional analysis using public databases from 10 countries worldwide, it was reported that the genus *Collinsella* could prevent severe illness after infection through bile acids [81]. The characteristic symptoms of COVID-19 are gastrointestinal disturbance, mood disorder, and extreme malaise, which persist for a long time after healing [82,83]. These are called long-term complications of COVID-19, and it has been suggested that these symptoms are related to dysbiosis [84,85,86,87,88].

Clarifying the relationship between the severity of COVID-19 and the microbiota is of great interest for performing therapeutic interventions [89]. Although microbiota studies need to solve the problem of difficulty in collecting stool specimens from patients with severe disease, in addition to a longitudinal observational analysis of the microbiota, it is desirable to elucidate the molecular mechanisms that trigger the rapid inflammatory state leading to cytokine storm formation. Cross-sectional data on alterations of the microbiota and pathogenesis of COVID-19 from various countries will also be of significant value.

## 5. Hepatitis Virus

### 5.1. Pathogenesis of Hepatitis Virus and Gut-Liver Axis

The intestinal tract and liver are anatomically and physiologically connected through the portal vein through the enterohepatic circulation system. Alterations in the gut microbiota are associated with viral hepatitis, mainly hepatitis B and C, and these affect the gut and liver through the so-called “enterohepatic axis”. It is becoming clear that gut microbiota-derived metabolites and cellular components affect the liver function through intestinal circulation and modulate the pathogenesis of liver diseases [90,91,92]. Hence, there is an indication of a crosstalk between the gut microbiota and the liver, and understanding this relationship is important, because it will help establish methods to control the gut bacteria for the treatment and prevention of liver diseases.

Currently, the specific mechanisms underlying the involvement of the components of the gut bacteria in viral hepatitis are not fully understood. However, the association between hepatitis and gut microbiota related to the enterohepatic axis has been studied extensively. It is suggested that various molecules, such as peptidoglycans and LPSs produced by the gut microbiota, are transferred to the liver, and the modification of bile acids by gut bacteria interacting with hepatic immune cells causes pathological effects [93]. For more information on the role of gut bacteria and abnormal bile acid secretion in liver dysfunction, future studies should assess the role of the gut bacteria and abnormal bile acid secretion in liver dysfunction in a more elaborated manner.

### 5.2. HAV and HEV Infection and Microbiota

Hepatitis A virus (HAV) and hepatitis E virus (HEV) are the causative agents of hepatitis A and E, respectively. Both HAV and HEV are excreted in the feces and spread through contaminated water and food. HAV can cause abdominal pain, vomiting, diarrhea, and fever, and 1% of infected individuals develop fulminant hepatitis, which can lead to acute renal failure. Owing to the epidemic and sporadic nature of HAV, only a few reports have examined its relationship with the intestinal microbiota. A fecal 16S rRNA analysis of the only reported HAV outbreak among Japanese HIV patients in 2017 showed significant dysbiosis immediately after acute hepatitis that persisted for a long time after the treatment [94].

The major symptoms of HEV infections are fever, malaise, myalgia, abdominal pain, and a skin rash. In rare cases, it can lead to acute liver failure. Like HAV, only a few reports have been published on the alteration of the intestinal microbiota in acute HEV infection. The composition of the microbiota is altered in patients with acute hepatitis E (AHE), compared with that in healthy subjects, and the abundance of some bacteria (Proteobacteria, Gammaproteobacteria, and Enterobacteriaceae) is related to the amount of interferon-gamma secreted [95]. In this report, the presence of Gammaproteobacteria was positively correlated with the serum alanine transaminase and total bilirubin levels, suggesting that the presence of Gammaproteobacteria may serve as an indicator to identify patients with AHE and to more accurately predict the severity. Additionally, a comparison between patients with hepatitis E and HEV-related acute liver failure showed that changes in the fecal microbiota were associated with HEV disease progression [96].

### 5.3. HBV Infection and Changes in the Intestinal Microbiota

Hepatitis B virus (HBV) infection is currently not curable, but it can be treated by suppressing viral replication and reducing complications. Five percent of adults develop chronic HBV infection, but approximately 90% of neonates and 30–50% of children under 5 years of age find it difficult to eliminate HBV from their body [97]. In addition to the possibility that maturation of the immune system causes an age-related difference in the HBV elimination ability, involvement of the intestinal microbiota is also speculated. Animal experiments suggested that anti-HBV activity is based on the regulation of the immune system by intestinal bacteria [98,99]. Several recent studies have shown a correlation between the intestinal microbiota and chronic HBV infection without liver injury [99,100,101,102], as well as HBV-related cirrhosis and hepatocellular carcinoma [103,104,105,106,107].

Chronic HBV infection without liver injury is associated with a decrease in bacteria of the genus *Bacteroides* [101,102], and multiple dysbiosis (although not consistently) of the gut microbiota is observed in HBV-related liver disease with disease progression. Several investigators attempted to use the operational taxonomic unit (OTU) profiles of these characteristic microbiota for their diagnosis [104,107,108]. However, in addition to regional differences, various factors such as alcohol intake, smoking, obesity, and race may influence the alteration of the gut microbiota, making the interpretation of the role of the gut microbiota in chronic hepatitis difficult. Furthermore, in HBV-infected individuals, a low HBV DNA copy number correlates with the diversity of the gut microbiota, with an *Alloprevotella*-rich microbiota profile being involved in lipid metabolism [109]. In the future, it will be important to understand the role of the gut microbiota in the interaction between HBV infection and the metabolic profile.

### 5.4. Efficacy of Early Treatment in the Recovery of Dysbiosis in HCV Infection 

Chronic HCV infection progresses to advanced hepatic fibrosis and cirrhosis in 20–30% of untreated patients, and hepatocellular carcinoma develops in 1–4% of patients [110]. Reports on the intestinal microbiota of patients with HCV infection mainly highlighted a decrease in the diversity of constituent species [111,112,113,114], while others reported an increase in diversity [51]. Like HBV infection, several differences, such as racial differences and stage of the disease, are suggested to affect the changes in the gut microbiota. Hepatitis C virus (HCV) RNA is detected in the blood, saliva, and bile of affected patients, as well as in their stool. This suggests a direct interaction between viral particles and the intestinal microbiota [115]. 

Direct-acting antiviral agents (DAAs) are currently used to treat HCV infection. They target the proteins of HCVs, inhibit their replication, and, consequently, eliminate them. A longitudinal analysis of the DAA treatment was recently conducted to improve the understanding of the association between hepatic HCV infection and the gut microbiota. It has been reported that the pattern of the microbiota at the beginning of the DAA treatment is related to its prognosis [116]. After treatment, the composition of the gut and oral microbiota in patients with mild fibrosis without cirrhosis was found to be similar to that of healthy individuals. Treatment-induced changes in the α-diversity were observed predominantly only in patients with a less advanced disease [117,118,119,120,121,122,123]. These results emphasize the importance of early therapeutic interventions for improving liver function by regulating the gut microbiota composition.

## 6. HIV

### 6.1. Chronic Inflammation with HIV Infection and Intestinal Microbiota

Despite advancements in antiretroviral therapy, the frequency of inflammation and immune activation remains elevated in patients with HIV infection, and these conditions are associated with morbidity and mortality [124,125]. The role of the gut microbiota in persistent HIV infection of the intestinal immune system is an interesting topic. However, the relationship between changes in the gut microbiota and persistent inflammation observed in patients remains unclear. Several studies demonstrated the possible role of the gut microbiota in influencing HIV infection through the rectal mucosa. A retrospective analysis using stool samples from men who have sex with men (MSM) during the acquired immunodeficiency syndrome pandemic suggested that the composition of the gut microbiota before HIV infection influenced the susceptibility to HIV-1 infection [126]. An intrarectal challenge with simian HIV in rhesus macaques showed that the more susceptible group had significantly more activated CCR5+ CD4+ T cells in the rectal mucosa, suggesting that differences in the gut microbiota are associated with differences in immune activation [127].

### 6.2. Two Trends in Gut Microbiota in HIV-Infected Patients

An alternative gut microbiota in HIV infection can potentially lead to or maintain systemic inflammation in patients. There are two major trends in the alteration of the gut microbiota in patients with HIV infection. The first trend is associated with sexual preference, which is independent of the HIV infection. In two independent European cohorts with different ethnic and cultural backgrounds, Noguera-Julian et al. found that MSM have a higher alpha diversity, i.e., increased and decreased number of bacteria of *Prevotella* and *Bacteroides*, respectively, than non-MSM, regardless of their HIV status [128]. This observation is consistent with the findings of several other studies on MSM [129,130]. Additionally, this trend was associated with receptive anal intercourse in both men and women [131]. The second reason is HIV infection. HIV-induced dysbiosis was associated with a reduced alpha diversity, enrichment of Gammaproteobacteria, including Enterobacteriaceae, and depletion of Lachnospiraceae and Ruminococcaceae, regardless of gender or sexual preference [131]. Increased or decreased frequencies of these bacteria were consistent with the findings of many other studies [132,133,134,135,136,137,138,139,140,141,142]. HIV-associated reduction of the alpha diversity was mainly observed in patients with low CD4 counts [143,144,145,146]. The restoration of the alpha diversity has been observed in some studies [146,147], while others have found no significant changes after antiretroviral therapy [134,135]. Enterobacteriaceae and Desulfovibrionaceae are also shown to be associated with various chronic inflammatory conditions, such as inflammatory bowel disease [148,149], suggesting a potential relationship between HIV-related dysbiosis and chronic inflammation. Further, several butyric acid-producing bacteria belonging to the Lachnospiraceae and Ruminococcaceae families were reduced in HIV-infected patients. Butyrate is an important energy source for colonic cells; it induces the expression of intestinal intercellular tight junction proteins that promote intestinal barrier function [150]. In patients with HIV, intestinal barrier dysfunction persists despite effective treatments, allowing the translocation of inflammatory microbial products into the systemic circulation [151]. 

### 6.3. Microbial Translocation and Systemic Inflammation

Chronic immune activation has been observed in HIV-1-infected patients even after treatment, suggesting the involvement of the intestinal microbiota. Indeed, many studies have demonstrated the association between intestinal dysbiosis and systemic inflammation [131,133,146,152,153,154]. The translocation of microorganisms and microbial products from the intestinal mucosa to the systemic circulation has been reported in HIV-infected individuals, and it is becoming clear that this is closely related to immune activation. An analysis of the simian immunodeficiency virus (SIV) reported that the progression to AIDS results in a decreased CD4 cell count and increased viremia, as well as the transfer of intestinal microbiota into the bloodstream [155]. Furthermore, in animal models of SIV infection, an early blockade of LPS entry from the intestinal tract into the blood circulation dramatically reduced T-cell activation and proliferation, inflammatory cytokine production, and the plasma SIV RNA levels [156]. There have been several reports showing a relationship between microbial translocation and chronic inflammation using blood markers that support the above observations [157,158,159]. Of these, intestinal fatty acid-binding protein, a marker of intestinal disorders, and sCD14, an indirect blood marker of microbial translocation in response to LPS, were associated in a cohort study of HIV-infected individuals [144,159]. These microbial translocation improvements have been shown to potentially affect immune recovery with treatment [160,161]. A 2-year follow-up study of HIV-infected treated patients reported that changes in the diversity and composition of translocated microbial species reduced the systemic inflammation and affect the CD4 recovery [161]. These observations suggest that microbiota regulation in parallel with therapeutic intervention may improve the clinical outcome.

In summary, the identification of microbiota that maintain chronic inflammation, which exacerbates HIV pathology, might be useful for the functional cure of HIV infection, which is currently difficult to eradicate. Moreover, elucidation of the molecular mechanisms leading to a leaky gut could be an effective therapeutic strategy, which includes control of the microbiota. Elucidation of the relationship between the characteristic microbiota profile of MSM and susceptibility to HIV infection is also important for knowledge in the prevention of HIV infection. 

## 7. HPV

### 7.1. HPV Infection and Unique Vaginal Bacterial Composition 

HPV is a DNA virus that infects more than 80% of sexually experienced women at least once during their lifetime. Most HPV infections heal spontaneously, but some persistent ones are responsible for the development of several diseases, including cervical, anal, and vaginal cancers and condyloma acuminatum. In addition to the role of HPV in carcinogenesis, the unique environment of individual mucosal sites, including mucosal secretions and the microbiota indigenous to the vagina, are likely to be involved in the persistence of infection and carcinogenesis of the HPV [162,163]. The microbiota in the vaginal mucosa is distinctive, and its role in HPV-induced carcinogenesis of the cervical epithelium has been extensively studied. 

### 7.2. Influence of Vaginal Bacterial Composition on HPV Infection

The composition of the human vaginal microbiota is classified into community state types (CSTs) based on the abundance of specific *Lactobacillus* species [164,165]. The vaginal microbiota is generally *Lactobacillus*-dominant but can also include *Lactobacillus crispatus* (CST I), *Lactobacillus gasseri* (CST II), *Lactobacillus iners* (CST III), and *Lactobacillus jensenii* (CST V). Women with vaginal microbiota comprising more diverse bacteria are considered to have a different CST (CST IV) [164]. Lactobacilli are thought to maintain the vaginal homeostasis directly in epithelial cells through the effects of their metabolites and indirectly by inhibiting the growth of pathogenic species [166]. Meanwhile, these CST patterns are associated with the risk of developing a disease. Women with CST IV have a higher risk of developing bacterial vaginosis (BV) than those with CST I [165,167]. Recent reports have shown that women with a *Lactobacillus*-deficient vaginal microbiota are at a higher risk of contracting sexually transmitted infections such as HIV infections than women with a *Lactobacillus*-dominant CST [168]. Furthermore, there was a significant decrease in *L. crispatus* in the vaginal microbiota of women with HPV infection and cervical intraepithelial tumors or invasive cervical cancer, and an *L. iners*-dominant vaginal microbiota profile was associated with a higher risk of cancer [169,170,171,172,173]. The local vaginal microbiota characteristic of cervical cancer caused by HPV infection is *Lactobacillus*-depleted and dominantly anaerobic [174]. Increased rates of HPV infection and persistence, as well as disease severity, are shown to be associated with vaginal microbial diversity and richness [175,176,177,178].

A decrease in lactate-producing lactobacilli leads to an increase in the vaginal pH (above 4.5), which, in turn, leads to bacterial overgrowth [179]. BV weakens the cervical epithelial barrier function and facilitates the entry of HPV into basal keratinocytes [180]. Furthermore, BV is associated with chronic inflammation at mucosal sites [181], suggesting that it may lead to the development of vaginal intraepithelial lesions. BV is caused by the rapid switching of lactic acid-producing bacteria in the vagina to several anaerobic bacteria, mainly *Gardnerella* [182]. *Gardnerella* has been reported to have a high detection rate in HPV-positive women; *Gardnerella* secretes sialidase (SNA), and its elevated concentration has been associated with the risk of cervical lesions [183,184]. A recent report also pointed out that SNA secreted by *Gardnerella* and *Prevotella* may play a critical role in HPV transmission to cervical lesions [185]. Additionally, mechanisms such as the transcriptional repression of Toll-like receptors by HPV-derived factors (E6/E7) allow HPV to escape immune elimination [186]. Recently, it has been reported that certain bacteriophages are associated with BV and that there is a BV-specific virome and microbiota. This indicates the importance of elucidating the interactions between bacteriophages and the bacterial trans-kingdom [187].

### 7.3. HPV Infection and Oral Microbiota in Oral Cancers 

Some oral cancers, such as oropharyngeal carcinoma (OPC), are known to be associated with HPV infection. The genus *Lactobacillus* is significantly present in saliva samples from patients with HPV-positive OPC [188,189], but the abundance and diversity of oral microbiota are low [188,190]. This decrease in diversity is in contrast with that of cervical cancer patients, suggesting the persistence of HPV and its impact on carcinogenesis by a few predominantly pathogenic bacteria. OPC patients have an abundance of endemic species of the vaginal microbiota, including *L. gasseri/johnsonii* and *L. vaginalis* [189]. The reason that these common vaginal bacterial species are found in the oral cavity is not clear, but transfer to the oral microbiota by sexual behavior is one possibility. Recent reports have documented changes in the diversity of the oral microbiota of HPV-infected individuals [191] and changes in the overall microbial community and bacterial abundance among HPV-positive OPC patients [192,193]. There have also been reports on the involvement of *Candida albicans* in carcinogenesis [194]. These observations suggest that the pathological progression caused by HPV infection may be due to the local environment caused by functional differences in the microbiota. It will be necessary to elucidate the molecular basis underlying the pathological progression caused by these changes in the microbiota.

## 8. Influence of Virome on Infectious Diseases

Humans have a huge microbiome, even while healthy, and not only have bacteria in their intestines but also viruses (DNA and RNA viruses) that infect prokaryotic cells and eukaryotic organisms [28,195]. Although the function of the viruses in the intestinal tract have not been investigated, bacteriophages that infect intestinal bacteria, which is the majority of enteric viruses, are thought to be involved in the regulation of the intestinal microbiota. This suggests that bacteriophages play an important role in intestinal homeostasis, including intestinal immunity of the host. Therefore, understanding the role of this phage would provide useful insights into the role of the gut microorganisms in infectious diseases. Although there have been several reports on phage research in viral infections, the causal relationship between phage research and the progression of infectious diseases has remained unclear [196].

Since HIV causes immunodeficiency, an increase in the opportunistic pathogens has long been reported, suggesting the presence of pathogenic viruses as the reason for its pathological progression. A 2016 report showing an association with pathogenesis reported a decreased CD4 T-cell count in the peripheral blood coupled with an elevated intestinal adenovirus (sequencing) and Enterobacteriaceae, contributing to AIDS-related intestinal disease and disease progression [143]. This was also confirmed in a mouse model of SIV, where vaccination against SIV was reported to inhibit the increase in intestinal viruses. This suggests that the weakening of the intestinal immunity has resulted in a loss of control of pathogens, which, in turn, has resulted in the spread of intestinal viruses [197]. This could be seen as an indicator of disease progression due to weakened immunity. Meanwhile, changes in several bacteriophages have been reported in HIV patients and COVID-19 patients [198,199,200,201,202,203,204,205]. Thus, elucidating the function of the virome on a viral infection will lead to an understanding of the pathophysiology of infectious diseases, although currently, it is not clear how this change is involved in the pathogenesis of disease progression. 

Until the development of advanced sequencing technologies, it has been difficult to gain a comprehensive understanding of infectious diseases in medical research by investigating the entire gut microorganism. It is hoped that future research focusing on the effect of trans-kingdom microbial interactions for pathogenesis will lead to advances in infectious disease research.

## 9. Approaches to the Study of the Intestinal Microbiota in Infectious Diseases

Recent advances in analytical techniques have improved our knowledge regarding the relationship between commensal bacteria and intestinal immunity. Microbiota–control interventions with probiotics [206] and fecal microbiota transplantation (FMT) may be effective in infectious diseases [207,208], but there have been reports of adverse events, and caution may be required [209], additionally, the differences in indigenous microbiota among nationalities must also be considered. Since interventional studies in humans are limited, in vitro and animal model studies may be useful for research and development [69,210,211,212]. HIV research includes a model of SIV infection using macaques, which has the potential to effectively assess the changes in the microbiota in disease progression and the role of the gut microbiota control in therapy effectiveness [213]. Analyses in mouse models are also promising, and a number of infectious disease studies have been conducted. It should be noted, however, that the use of mice in a more natural environment may yield results more similar to human physiology, unlike certain mice bred in the laboratory [15,214,215]. Much of the research on microbial–viral interactions remains correlational. The causal relationships remain unclear, as viral infections can affect the microbiota. Therefore, animal models and in vitro experimental systems may also be useful tools in understanding these causal relationships and the underlying molecular mechanisms, which have been reviewed extensively elsewhere [216,217]. In addition, it is necessary to elucidate the molecular mechanism of the immune activation of secondary metabolites produced by bacteria and clarify the role of molecules such as extracellular vesicles [218], whose functions have recently been explained as communication tools among bacteria and between bacteria and host cells, in infectious diseases.

## 10. Conclusions and Future Perspectives

In this review, we outlined viral infections based on three foci: viral infection, target tissues, and the surrounding environment, including the indigenous microbiota of the target tissues. Viral infections can be divided into transient infections, such as upper respiratory tract infections and gastroenteritis, and chronic persistent infections, such as hepatitis and HPV and HIV infections. Transient infections cause abnormalities of the microbiota as part of the immune response; however, in mild cases, the balance of the microbiota is restored over time. However, if some microorganisms of the microbiota are imbalanced, then systemic inflammation may be triggered. It is necessary to identify the bacteria involved in this. Based on reports of infections, such as those on COVID-19, the normal microbiota profile before infection may have a significant impact on its prognosis. This is probably true for all viral infections, as it has been suggested that the bacterial profile indigenous to the vagina is also involved in the pathogenesis and development of a HPV infection. Further, persistent infections can lead to chronic inflammation and prolonged dysbiosis. Tissue exhaustion and chronic inflammatory conditions gradually facilitate the progression of the pathological process, and this is relatively slow. This temporal concept has contributed to the difficulty in interpreting the involvement of the microbiota, but it is necessary to clarify the details of their involvement using animal and in vitro experiments. The number of reports on the involvement of the microbiota in viral infections is increasing, but because of the limitations of this review, which covers a wide range of viruses, the contents of this paper are limited to a summary report including the main points. 

Furthermore, phages infecting the microbiota could be utilized for new interventions that act by regulating the microbiota. A microbiota analysis tends to be an observational study, but it is also important to elucidate the mechanisms of virus–host–microbiota interactions at the molecular level. The analysis of the role of fungi in disease progression is also important and should be reported more frequently [219]. Elucidating the role of symbiotic bacterial species, their coexistence with humans, and their interactions at the molecular and genetic levels will help advance our knowledge of infectious diseases. This could be useful in viral infections, as well as for many other diseases.

## Data Availability

Not Applicable.
